# New disinfection and sterilization methods.

**DOI:** 10.3201/eid0702.010241

**Published:** 2001

**Authors:** W A Rutala, D J Weber

**Affiliations:** University of North Carolina (UNC) Health Care System and UNC School of Medicine, Chapel Hill, North Carolina, USA. brutala@unch.unc.edu

## Abstract

New disinfection methods include a persistent antimicrobial coating that can be applied to inanimate and animate objects (Surfacine), a high-level disinfectant with reduced exposure time (ortho-phthalaldehyde), and an antimicrobial agent that can be applied to animate and inanimate objects (superoxidized water). New sterilization methods include a chemical sterilization process for endoscopes that integrates cleaning (Endoclens), a rapid (4-hour) readout biological indicator for ethylene oxide sterilization (Attest), and a hydrogen peroxide plasma sterilizer that has a shorter cycle time and improved efficacy (Sterrad 50).


### New Disinfection and Sterilization Methods

**William A. Rutala and David J. Weber**
University of North Carolina (UNC) Health Care System and
UNC School of Medicine, Chapel Hill, North Carolina, USA

New disinfection methods include a persistent antimicrobial coating that can be applied to inanimate and animate objects (Surfacine), a high-level disinfectant with reduced exposure time (ortho-phthalaldehyde), and an antimicrobial agent that can be applied to animate and inanimate objects (superoxidized water). New sterilization methods include a chemical sterilization process for endoscopes that integrates cleaning (Endoclens), a rapid (4-hour) readout biological indicator for ethylene oxide sterilization (Attest), and a hydrogen peroxide plasma sterilizer that has a shorter cycle time and improved efficacy (Sterrad 50).

The need for appropriate disinfection procedures is highlighted by the multitude of outbreaks resulting from improperly decontaminated patient-care items. Because sterilizing all such items is unnecessary, hospital policies need to identify whether cleaning, disinfection, or sterilization is indicated based primarily on an item's intended use but considering other factors including cost. We review new methods of disinfection and sterilization. Criteria for inclusion were technologies cleared in 1999 or 2000 by the Food and Drug Administration (FDA) or submitted to the FDA or Environmental Protection Agency (EPA) but not yet cleared (Table 1). These technologies have the potential to improve patient care, but in general their antimicrobial activity has not been independently validated.

Table 1. New methods in disinfection and sterilization

| Process | Agent | Regulatory agency action |
|---|---|---|
| Disinfection | Ortho-phthalaldehyde (Cidex OPA) | FDA cleared, October 1999 |
| | Antimicrobial coating (Surfacine) | Not FDA/EPA cleared |
| | Superoxidized water (Sterilox) | Not FDA/EPA cleared |
| Sterilization | Liquid sterilization process (Endoclens) | Not FDA cleared |
| | Rapid readout ethylene oxide biological indicator (Attest) | Not FDA cleared |
| | New plasma sterilizer (Sterrad 50) | FDA cleared, Jan 1999 |

### Rational Approach to Disinfection and Sterilization

More than 25 years ago, Spaulding devised an approach to disinfection and sterilization of patient-care items or equipment that has proved to be so clear and logical that it has been retained, refined, and successfully used by infection control professionals (1). Spaulding believed that how an object should be disinfected depended on its intended use. The three categories he described were critical, semicritical, and noncritical. Critical objects (those that enter sterile tissues or the vascular system or through which blood flows, such as implanted medical devices) should be sterile when used. Semicritical items (that touch mucous membranes or nonintact skin, e.g., endoscopes, respiratory therapy equipment, and diaphragms) require high-level disinfection (i.e., elimination of all microorganisms except high numbers of bacterial spores). Noncritical items (bedpans, blood pressure cuffs, and bedside tables) require only low-level disinfection.

Address for correspondence: William A. Rutala, 547 Burnett-Womack, CB #7030, Division of Infectious Diseases, UNC at Chapel Hill, Chapel Hill, NC 27599-7030; fax: 919-966-6714; e-mail: brutala@unch.unc.edu

### Ortho-phthalaldehyde: A New Chemical Sterilant

Ortho-phthalaldehyde (OPA) received clearance by FDA in October 1999. OPA solution is a clear, pale-blue liquid (pH 7.5), which typically contains 0.55% OPA. OPA has demonstrated excellent microbiocidal activity in in vitro studies (2,3). For example, it has shown superior mycobactericidal activity (5-$\log_{10}$ reduction in 5 minutes) compared with glutaraldehyde. The mean time required to effect a 6-$\log_{10}$ reduction for *M. bovis* using 0.21% OPA was 6 minutes, compared with 32 minutes using 1.5% glutaraldehyde (Table 2) (4). When tested against a wide range of microorganisms, including glutaraldehyde-resistant mycobacteria and *Bacillus subtilis* spores (5), OPA showed good activity against the mycobacteria tested, including the glutaraldehyde-resistant strains, but 0.5% OPA was not sporicidal within 270 minutes of exposure. Increasing the pH from its unadjusted level (about 6.5) to pH 8 improved sporicidal activity.

OPA has several potential advantages compared with glutaraldehyde. It requires no activation, is not a known irritant to the eyes and nasal passages, has excellent stability over a wide range of pH (pH 3-9), does not require exposure monitoring, and has a barely perceptible odor. Like

Table 2. Activity of glutaraldehyde and ortho-phthalaldehyde against *Mycobacterium bovis*

| Disinfectant | Time for 6-$\log_{10}$ reduction[a] |
|---|---|
| 1.5% glutaraldehyde | 28-36 minutes |
| 2.5% glutaraldehyde | 14-18 minutes |
| 0.21% ortho-phthalaldehyde | 4.8-6.3 minutes |

[a]Range of values from two different laboratories (4).

glutaraldehyde, OPA has excellent material compatibility. A potential disadvantage is that OPA stains proteins gray (including unprotected skin) and thus must be handled with caution (i.e., use of gloves, eye protection, fluid-resistant gowns when handling contaminated instruments, contaminated equipment, and chemicals) (2,3). Limited clinical studies of OPA are available. In one clinical-use study of 100 endoscopes exposed for 5 minutes to OPA, a $\geq$ 5-$log_{10}$ reduction in bacterial load occurred, and OPA was effective over a 14-day usage cycle (6). Manufacturer's data show that OPA will last longer before reaching its minimum effective concentration limit (about 82 cycles) compared with glutaraldehyde (after 40 cycles) in an automatic endoscope reprocessor (7). Disposal must be in accordance with local and state regulations. If OPA disposal in the sanitary sewer is restricted, glycine (25 g/gallon) can be used to neutralize the OPA and make it safe for disposal.

The high-level disinfectant label claims for OPA solution at 20°C vary: 5 minutes in Europe, Asia, and Latin America; 10 minutes in Canada; and 12 minutes in the United States. FDA clearance was based on a "simulated-use" test requirement for a 6-$log_{10}$ reduction of resistant bacteria suspended in organic matter and dried onto an endoscope. Since this test does not include cleaning, an essential component of disinfection of reusable devices (e.g., endoscopes), it is likely that the time required for high-level disinfection of a medical device by OPA would be less than 12 minutes. Efficacy test results using mycobacteria support a 5-minute exposure time at room temperature for OPA with a greater than 5-$log_{10}$ reduction. Canadian regulatory authorities require a 6-$log_{10}$ reduction in mycobacteria (this requires approximately 6 min) and allow only 5-minute exposure time intervals; thus, the exposure time for Canadians was set at 10 minutes (CG Roberts, pers. commun., Feb 2000).

### Surfacine: A New Antimicrobial Agent

Contaminated environmental surfaces have been associated with transmission of certain nosocomial pathogens, principally vancomycin-resistant *Enterococcus* spp. (VRE), methicillin-resistant *Staphylococcus aureus* (MRSA), and *Clostridium difficile*. The incidence of nosocomial infections caused by VRE in particular has dramatically increased in the past decade. Cross-transmission is thought to result from transient hand carriage by hospital personnel, who may potentially be colonized directly from contact with colonized or infected patients or indirectly by contact with a contaminated environmental surface. Cultures of surfaces in rooms of patients colonized or infected with VRE have yielded positive cultures in 7% to 37% of samples. Molecular analysis of VRE strains involved in outbreaks has in some cases demonstrated that isolates obtained from the environment were identical to the outbreak strain (8).

Antibiotic-resistant pathogens such as VRE and MRSA possess similar susceptibility to disinfectants as antibiotic-susceptible strains (9,10). However, commonly used surface disinfectants such as phenols and quaternary ammonium compounds, while effective in eliminating these pathogens, do not have residual activity. Hence, after disinfection, surfaces may rapidly be recontaminated.

Surfacine is a new, persistent antimicrobial agent that may be used on animate or inanimate surfaces. It incorporates a water-insoluble antimicrobial compound (silver iodide) in a surface-immobilized coating (a modified polyhexamethylenebiguanide) that is capable of chemical recognition and interaction with the lipid bilayer of the bacterial outer cell membrane by electrostatic attraction. The intimate microbial contact with the surface results in transfer of the antimicrobial component (silver) directly from the coating to the organism. Microorganisms contacting the coating accumulate silver until the toxicity threshold is exceeded; dead microorganisms eventually lyse and detach from the surface. The amount of silver present and the number of microorganisms in contact with the treated surface determine how long the coating is effective. Preliminary studies show that treated surfaces result in excellent elimination of antibiotic-resistant bacteria (e.g., VRE) inoculated directly on various surfaces at challenge levels of 100 CFU/sq inch for at least 13 days (Table 3) (11). Antimicrobial activity is retained when the surface is subjected to repeated dry wiping or wiping with a quaternary ammonium compound. Data available from the manufacturer demonstrate inactivation of bacteria, yeast, fungi, and viruses when the product is applied at challenge levels of up to $10^6$ CFU/mL. Sustained antimicrobial activity has been shown for the tested microorganisms. Inactivation times for microorganisms vary.

This persistent antimicrobial agent transfers the active biocide (silver) "on demand" directly to the organism without elution of silver ions into solution. The coating, therefore, functions in a chemically intelligent way, i.e., antimicrobial response is triggered only upon microbial contact. The mechanism of silver release differs from that of conventional, topically applied silver compounds (e.g., silver nitrate and silver sulfadiazine), which work by generating a bactericidal level of silver ions. (The ions are released into aqueous solution either by silver oxide or dissolution of the silver salt.)

This new antimicrobial agent can be applied to animate and inanimate surfaces by dipping, brushing, or spraying without prior surface treatment. The coating does not undergo photoreduction, degradation, or color change when exposed to intense UV irradiation (4 mW/$cm^2$ for 2 hr). This new antimicrobial agent has excellent adhesion to virtually all substrates, is optically clear, and does not delaminate, flake, or crack. Treated surfaces subjected to a wipe test retained their antimicrobial efficacy (Table 3) (11). Permanently treated surfaces remained chemically inert and retained their biocidal activity after exposure to various physical and chemical stresses such as temperature (tested from –20°C to 130°C), solvents (alcohol), solutions with a pH of 4 to 10, solutions of high ionic strength, and sterilization by conventional methods (e.g., steam, ethylene oxide, gamma-irradiation). The coating contains low levels of silver iodide (approx. 10 µg/$cm^2$ of coated surface), and coated surfaces are resistant to biofilm formation. Surfacine does not cause mammalian cell toxicity and passes the acute systemic toxicity tests recommended by the U.S. Pharmacopeia (SP Sawan and S Subramanyan, pers. commun., 2000).

Table 3. Effect on vancomycin-resistant Enterococcus (VRE) survival of wiping Surfacine on a treated surface over an extended period

| Surface | Intervention | Day 1 | Day 6 | Day 13 |
|---|---|---|---|---|
| Formica | Control | 50 | 95 | 120 |
| | Treated | 0 (100%)[a] | 0 (100%) | 0 (100%) |
| | Treated & wiped | 0 (100%) | 0 (100%) | 0 (100%) |

[a]Percent reduction of VRE counts per Rodac plate ([treated/control] x 100) (11).

If novel surface treatments such as this product prove to be effective in significantly reducing microbial contamination, are cost-effective, and have long-term residual activity, they may be extremely useful in limiting transmission of nosocomial pathogens. The antimicrobial activity of this coating makes it potentially suitable for a wide range of applications, including disinfection of surfaces, microporous filters, and medical devices and use as a topical ointment or hand antiseptic.

### A New Disinfectant: Superoxidized Water

The concept of electrolyzing saline to create a disinfectant is appealing because the basic materials, saline and electricity, are cheap and the end product (water) is not damaging to the environment. A commercial adaptation of this process, Sterilox, is available in the United Kingdom. The mode of action is not clear but probably relates to a mixture of oxidizing species. The main products are hypochlorous acid at a concentration of approximately 144 mg/L and free chlorine radicals. This disinfectant is generated at the point of use by passing a saline solution over titanium-coated electrodes at 9 amps. The product generated has a pH of 5.0-6.5 and an oxidation reduction potential of >950 mV. Equipment to produce the product may be expensive because parameters such as pH, current, and redox potential must be closely monitored. The solution has been shown to be nontoxic to biological tissues. Although the solution is claimed to be noncorrosive and nondamaging to endoscopes, one flexible endoscope manufacturer has voided the warranty on its endoscopes because superoxidized water was used to disinfect them (12).

The antimicrobial activity of this new sterilant has been tested against bacteria, mycobacteria, viruses, fungi, and spores (13-15). Recent data have shown that freshly generated superoxidized water is rapidly effective (<2 minutes) in achieving a 5-$\log_{10}$ reduction of pathogenic microorganisms (*Mycobacterium tuberculosis*, *M. chelonae*, poliovirus, HIV, MRSA, *Escherichia coli*, *Candida albicans*, *Enterococcus faecalis*, *Pseudomonas aeruginosa*) in the absence of organic loading. However, the biocidal activity of this disinfectant was substantially reduced in the presence of organic material (5% horse serum) (14). Additional studies are needed to determine if this solution may be used as an alternative to other disinfectants.

### Endoclens: A New Liquid Chemical Sterilization System

A new automated endoscope-reprocessing system has been submitted to FDA for clearance. The system is designed to provide rapid, automated, point-of-use chemical sterilization of flexible endoscopes and consists of a computer-controlled endoscope-reprocessing machine and a new, proprietary liquid sterilant that uses performic acid. The sterilant is produced, as needed by the machine, by automatic mixing of the two component solutions of hydrogen peroxide and formic acid. This sterilant is fast-acting against spore-forming bacteria (Table 4). The system's major features are an automatic cleaning process, capability to process two flexible scopes asynchronously, automated channel blockage and leak detection, filter water rinsing and scope drying after sterilization, hard-copy documentation of key process parameters, user-friendly machine interface, and total cycle time less than 30 minutes. The reprocessor can also be disinfected automatically to prevent infection or pseudoinfection.

Table 4. Activity of performic acid against spore-forming bacteria[a]

| | Lot 1 | Lot 2 |
|---|---|---|
| *Bacillus subtilis*[b] | 0/30 growth | 0/30 growth |
| *B. subtilis*[c] | 0/30 growth | 0/30 growth |
| *Clostridium sporogenes*[b] | 0/30 growth | 0/30 growth |
| *C. sporogenes*[c] | 0/30 growth | 0/30 growth |

[a]Methodology: AOAC Sporicidal Activity Test, 10-min exposure; 1800 ± 500 ppm performic acid; hard water/aged starting solution at 44 ±2°C.
[b]Silk sutures.
[c]Porcelain cylinders.

The reprocessor can independently process two endoscopes at the user's discretion since it has two washing/sterilization bays. The endoscopes are attached to special holders (racks), which slide into the machine bays located in the front of the machine and provide a connection between the reprocessor and the endoscope's inner channels. The endoscope racks are designed to accommodate all types of flexible endoscopes. During washing, enzymatic detergent is automatically dispensed, diluted with warm water (45°C), and sprayed onto the exterior endoscope surfaces and pumped through the endoscope lumens. The enzymatic detergent is pumped through the lumens with alternating pulses of compressed air to assist in removing any adhering material. Cleaning studies performed by the manufacturer using a synthetic soil show the system can satisfactorily clean and rinse detergents from an endoscope in preparation for point-of-use sterilization.

The concentration and temperature of the mixed chemicals are automatically measured by the machine with refraction and temperature sensors. Once pumped into the washing/sterilization bay, the sterilant is vigorously sprayed over all exterior endoscope surfaces and pumped through all endoscope lumens to sterilize the scope. Simulated-use studies with resistant spores suspended in 5% serum and inoculated on scope surfaces and inside lumens have demonstrated the effectiveness of the sterilant.

All water used for washing/sterilization and rinsing is filtered through a 0.2-µm filter. The scopes are dried when the cycle is completed by using filtered compressed air that is sprayed over the exterior scope surfaces and through the interior lumens through the same connections used for the washing and sterilization steps.

The total cycle time for scope testing, washing, sterilization, and drying is less than 30 minutes. Upon completion of each cycle, the reprocessor prints a hard-copy record as well as retaining a record in memory, accessible through its floppy disk drive. Printer parameters are printed at the completion of each cycle and include scope identification, processing date, key cycle parameters, space for insertion of patient name or identification number, procedure type, and date (16; CG Roberts, pers. commun., 2000).

### Attest Ethylene Oxide (EO) Rapid Readout

EO has been widely used as a low-temperature sterilant since the 1950s. It is the most commonly used process for sterilizing temperature- and moisture-sensitive medical devices and supplies in U.S. health-care institutions. Until

December 1995, EO sterilizers were combined with a chlorofluorocarbon stabilizing agent, but these agents were phased out because they were linked to destruction of the earth's ozone layer. Alternative technologies currently available and cleared by FDA include 100% EO and EO with different stabilizing gases, such as carbon dioxide ($CO_2$) or hydrochlorofluorocarbon (17). A new rapid readout EO biological indicator, designed for rapid and reliable monitoring of EO sterilization processes, is available outside the United States but has not yet been cleared by FDA.

Sterilization (the complete elimination or destruction of all forms of microbial life) is recommended for all "critical" medical items, such as surgical instruments, cardiac and urinary catheters, implantable devices (e.g., heart valves), and needles. Because it is essential to ensure sterilization of critical items, monitoring of the sterilization process is advised. Monitors may be mechanical, chemical, or biological. Biological monitors are recommended because, unlike chemical indicators, they measure the sterilization process directly by using the most resistant microorganism (e.g., *B. subtilis*), not by merely testing the physical and chemical conditions necessary for sterilization (18,19).

The new rapid readout EO biological indicator will indicate an EO sterilization process failure by producing a fluorescent change, which is detected in an auto-reader within 4 hours of incubation at 37°C, and a visual pH color change of the growth media within 96 hours of continued incubation. The rapid readout EO biological indicator detects the presence of *B. subtilis* by detecting the activity of an enzyme present within the *B. subtilis* organism, beta-glucosidase. The fluorescence indicates the presence of active spore-associated enzyme and a sterilization process failure. The rapid readout EO biological indicator also detects acid metabolites produced during growth of the *B. subtilis* spore. The acid metabolites are the result of a series of enzyme-catalyzed reactions that occur during spore growth. The growth produces a pH change in the medium that causes the medium to change color from green to yellow, indicating an EO sterilization process failure.

For hospital use, a monitor should be easy to use, inexpensive, and not subject to exogenous contamination; provide positive results as soon as possible after the cycle so that corrective action may be taken; and provide positive results only when the sterilization parameters (e.g., EO concentration, humidity, time, temperature) are adequate to kill microbial contaminants. However, the biological indicator should not be so resistant that it causes needless recall and overprocessing (18). The rapid readout EO biological indicator has potential for substantially improving assessment of EO cycles. According to manufacturer's data, the enzyme was always detected whenever viable spores were present. This was expected because the enzyme is relatively EO resistant and is inactivated at a slightly longer exposure time than the spore.

The rapid readout EO biological indicator can be used to monitor 100% EO, EO-chlorofluorocarbons, and EO-hydrochlorofluorocarbon mixture sterilization cycles. It has not been tested in EO-$CO_2$ mixture sterilization cycles. The self-contained design (i.e., it contains both the spore strip and growth media) of the indicator makes it easy to use in the department where the sterilizer is located. The rapid readout EO biological indicator should be placed in a test pack (e.g., the Association for the Advancement of Medical Instrumentation) and placed in a full sterilizer load in the most challenging area for the sterilizer (for EO placement should be in the center). Data show that the 4-hour fluorescent sensitivity of this indicator is $\geq$ 97%, on the basis of the number of visual growth-positive indicators after 168 hours (7 days) of incubation at 37°C. In fact, all the 7-day growth-positive indicators were detected by fluorescence within 4 hours of incubation (Table 5), indicating that if there is no fluorescence at 4 hours, no growth-positive indicators will be detected with continued incubation.

The ability to monitor EO cycles in a surgical suite or central processing and to have results in 4 hours should enable operating room staff to intercept improperly sterilized items either before use or before a surgery ends. If a hospital could quarantine the load for the 4-hour readout, the need for recalls of potentially nonsterile packages and for informing physicians about the use of nonsterile medical devices could be eliminated. New indicator technologies such as the rapid readout EO biological indicators are likely to improve patient safety (20, PM Schneider, pers. commun., 2000).

Table 5. Sensitivity of Attest rapid readout ethylene oxide biological indicator

| Sterilization process | Incubation temp. (°C) | No. tested | No. growth positives (168 hr) | False-negatives (4 hr) | Sensitivity (4 hr) |
|---|---|---|---|---|---|
| 37°C 600 mg EO/L, 60% relative humidity | 37 | 1,100 | 752 | 0 | 100% |
| 54°C 600 mg EO/L, 60% relative humidity | 37 | 1,300 | 842 | 0 | 100% |

### A New Low-Temperature Sterilization Technology: Hydrogen Peroxide Plasma

Alternative technologies to sterilize temperature-sensitive equipment are being developed. A new hydrogen peroxide plasma sterilizer, the Sterrad 50, was recently cleared by FDA. It is a smaller version (44-L sterilization chamber) of the Sterrad 100 (73-L sterilization chamber), cleared in 1991. The Sterrad 50 contains a single shelf for placement of instruments to be sterilized within a rectangular chamber, whereas the Sterrad 100 has two shelves and a cylindrical chamber. The operational design of the two sterilizers is similar except that the Sterrad 50 consists of two hydrogen peroxide vapor-diffusion stage-plasma cycles. The sterilization cycles of the Sterrad 50 and Sterrad 100 are 45 minutes and 72 minutes, respectively.

The Sterrad 50 was equally as effective as EO in killing approximately $10^6$ *B. stearothermophilus* spores present in the center of narrow-lumen stainless steel tubes (Table 6).

Table 6. Comparative evaluation of sporicidal activity of new low-temperature sterilization technologies (21,22)

| Sterilization method | Units positive/units tested | | | |
|---|---|---|---|---|
| | LTU,[a] 3 mm | LTU 2 mm | LTU 1 mm | SL,[b] 3 mm |
| EO-HCFC | 0/50 | 0/40 | 0/40 | 0/50 |
| Sterrad 100S | 0/50 | 0/40 | 0/40 | 0/40 |
| Sterrad 50 | 0/30 | 0/30 | 0/30 | 0/30 |
| Sterrad 100 | 2/40 | 3/40 | 37/50 | 0/40 |

[a]LTU = lumen test unit.
[B]SL = straight lumen.

The Sterrad 50 and EO sterilized the carriers in even the smallest-lumened device, which was 1 mm in diameter (21).

### Conclusions

New sterilization and disinfection technologies may provide significant advantages over existing technologies (Table 7). However, data currently available have primarily been generated by the manufacturers and need to be independently validated. If these new technologies are demonstrated to be effective, their cost-effectiveness compared with standard technologies should be assessed. These new technologies hold the promise of improved patient care.

Dr. Rutala is director of the Hospital Epidemiology, Occupational Health and Safety Program at the University of North Carolina (UNC) Health Care System and professor of medicine at UNC School of Medicine, Chapel Hill, NC. His research interests include prevention of nosocomial infections, disinfection, and sterilization.

Dr. Weber is medical director of the Hospital Epidemiology, Occupational Health and Safety Program at UNC Health Care System and professor of medicine, epidemiology, and pediatrics at the UNC Schools of Medicine and Public Health. His research interests include prevention of nosocomial infections, disinfection, and sterilization.

Table 7. Comparison of new and standard disinfection and sterilization technologies

| Technology | | Comparison of new with standard technology | | |
|---|---|---|---|---|
| New | Standard | Advantages | Disadvantages | Future needs |
| OPA | Glutaraldehyde | -Shorter process time (12 vs. 45 min)<br>-No activation<br>-Not a known irritant to eyes and nasal passages<br>-No vapor ceiling limit<br>-Weak odor | -Stains protein gray<br>-Higher cost | -Additional studies of antimicrobial efficacy<br>-Cost-effectiveness study<br>-Study of effectiveness in actual clinical use<br>-Verification of more cycles per solution than glutaraldehyde |
| Surfacine | Disinfectants (phenolics quaternary ammonium); Antiseptics (alcohol, iodophor, chlorhexidine gluconate) | -Antimicrobial persistence (>13 days)<br>-May be used on animate and inanimate surfaces<br>-Broad antimicrobial spectrum<br>-Transfers active agent (silver) to microbes on demand without elution<br>-Resistant to forming biofilm<br>-No toxicity to mammalian cells | -Cost? | -Assess microbicidal activity against broad spectrum of pathogens<br>-Demonstration of efficacy to reduce nosocomial infections<br>-Human safety and toxicity data for use as an antiseptic<br>-Demonstrate antimicrobial activity in presence of organic matter |
| Super-oxidized water | High- or low-level disinfectants; antiseptics | -Basic materials (saline and electricity) inexpensive<br>-End product not damaging to environment<br>-Nontoxic to biological tissues | -Production equipment expensive due to monitoring<br>-Endoscope compatibility unknown<br>-Decreased efficacy in presence of organic matter<br>-Limited-use life (must be freshly generated) | -Evaluation of endoscope compatibility<br>-Cost-effectiveness study |
| Endoclens | None | -Device automatically cleans and sterilizes<br>-Rapid cycle time (<30 min)<br>-Tests endoscope for channel blockage and leaks<br>-Advantages of automated process (e.g., consistent exposure to sterilant, filtered water rinse, operator convenience) | -Cost?<br>-Used for immersible instruments only<br>-Point-of-use system, no long-term storage | -Cost-effectiveness study<br>-Study of effectiveness in actual clinical use<br>-Assessment of microbicidal activity |
| EO rapid readout | 48-hr spore readout biological indicator | -Rapid (4-hr), reliable assessment of sterilization efficacy<br>-Prevents recall of released sterilization loads | -Cost?<br>-Not tested with EO and $CO_2$ mixtures | -Cost-effectiveness study<br>-Validation of claimed 100% sensitivity |
| Plasma sterilizer | Hydrogen peroxide gas plasma sterilizer | -Use of two hydrogen peroxide diffusion-plasma stage cycles is a more effective sterilization process<br>-Reduced cycle time (45 min)<br>-Various sized units available<br>-Leaves no toxic residues | -Cost?<br>-Endoscopes with lengths >40 cm or a diameter of <3 mm cannot be processed | -Cost-effectiveness study<br>-Study of effectiveness in actual clinical use |

### References

1. Rutala WA, APIC Guidelines Committee. APIC guideline for selection and use of disinfectants. Am J Infect Control 1996;24:313-42.
2. Rutala WA, Weber DJ. Disinfection of endoscopes: review of new chemical sterilants used for high-level disinfection. Infect Control Hosp Epidemiol 1999;20:69-76.
3. Advanced Sterilization Products, Johnson & Johnson. Cidex OPA high level disinfection solution: technical information. Irvine (CA): Advanced Sterilization Products; 1999.
4. Gregory AW, Schaalje B, Smart JD, Robison RA. The mycobactericidal efficacy of ortho-phthalaldehyde and the comparative resistances of *Mycobacterium bovis*, *Mycobacterium terrae*, and *Mycobacterium chelonae*. Infect Control Hosp Epidemiol 1999:20:324-30.
5. Walsh SE, Maillard JY, Russell AD. Ortho-phthalaldehyde: a possible alternative to glutaraldehyde for high level disinfection. J Appl Microbiol 1999;86:1039-46.
6. Alfa MJ, Sitter DL. In-hospital evaluation of ortho-phthalaldehyde as a high level disinfectant for flexible endoscopes. J Hosp Infect 1994;26:15-26.
7. Chen X. A comparison of the useful life of Cidex activated dialdehyde solution and Cidex OPA in AER system. Irvine (CA): Advanced Sterilization Products; 1999.
8. Weber DJ, Rutala WA. Role of environmental contamination in the transmission of vancomycin-resistant enterococci. Infect Control Hosp Epidemiol 1997;18:306-9.
9. Rutala WA, Stiegel MM, Sarubbi FA, Weber DJ. Susceptibility of antibiotic-susceptible and antibiotic-resistant hospital bacteria to disinfectants. Infect Control Hosp Epidemiol 1997;18:417-21.
10. Anderson RL, Carr JH, Bond WW, Favero MS. Susceptibility of vancomycin-resistant enterococci to environmental disinfectants. Infect Control Hosp Epidemiol 1997;18:195-9.
11. Rutala WA, Gergen MF, Weber DJ. Evaluation of a new surface germicide (Surfacine™) with antimicrobial persistence. Infect Control Hosp Epidemiol 2000;21:103.
12. Fraise AP. Choosing disinfectants. J Hosp Infect 1999;43:255-64.
13. Tanaka H, Hirakata Y, Kaku M, Yoshida R, Takemura H, Mizukane R, et al. Antimicrobial activity of superoxidized water. J Hosp Infect 1996;34:43-9.
14. Selkon JB, Babb JR, Morris R. Evaluation of the antimicrobial activity of a new super-oxidized water, Sterilox®, for the disinfection of endoscopes. J Hosp Infect 1999;41:59-70.
15. Shetty N, Srinivasan S, Holton J, Ridgway GL. Evaluation of microbicidal activity of a new disinfectant: Sterilox® 2500 against *Clostridium difficile* spores, *Helicobacter pylori*, vancomycin resistant *Enterococcus* species, *Candida albicans* and several *Mycobacterium* species. J Hosp Infect 1999;41:101-5.
16. Advanced Sterilization Products new automated endoscope reprocessing system with NSX sterilant. Technical report. Irvine (CA): Advanced Sterilization Products; 2000.
17. Rutala WA, Weber DJ. Clinical effectiveness of low-temperature sterilization technologies. Infect Control Hosp Epidemiol 1998;19:798-804.
18. Rutala WA, Gergen MF, Weber DJ. Evaluation of a rapid readout biological indicator for flash sterilization with three biological indicators and three chemical indicators. Infect Control Hosp Epidemiol 1993;14:390-4.
19. Rutala WA, Jones SM, Weber DJ. Comparison of a rapid readout biological indicator for steam sterilization with four conventional biological indicators and five chemical indicators. Infect Control Hosp Epidemiol 1996:17:423-8.
20. 3M Attest Rapid Readout Ethylene Oxide Biological Indicator. Product Profile. Minneapolis (MN): 3M.
21. Rutala WA, Gergen MF, Weber DJ. Sporicidal activity of a new low-temperature sterilization technology: the Sterrad 50 sterilizer. Infect Control Hosp Epidemiol 1999; 20:514-16.
22. Rutala WA, Gergen MF, Weber DJ. Comparative evaluation of the sporicidal activity of new low-temperature sterilization technologies: ethylene oxide, 2 plasma sterilization systems, and liquid peracetic acid. Am J Infect Control 1998;26:393-8.
